# North and South in Medieval Iberia: A historical and environmental estimate through isotopic analyses

**DOI:** 10.1371/journal.pone.0304313

**Published:** 2024-06-05

**Authors:** José Francisco Martín-Alonso, Zita Laffranchi, Marco Milella, Lorenza Coppola-Bove, Luis A. Mena-Sánchez, Sylvia A. Jiménez-Brobeil

**Affiliations:** 1 Department of Legal Medicine, Toxicology and Physical Anthropology, University of Granada, Granada, Spain; 2 Department of Physical Anthropology, Institute of Forensic Medicine, University of Bern, Bern, Switzerland; Universidad de Sevilla, SPAIN

## Abstract

The Middle Ages in the Iberian Peninsula is a period of special interest for studying the relationship of climate change with historical and socioeconomic processes. Between the 8^th^ and 15^th^ centuries AD, the Peninsula was characterized not only by complex political, cultural, and social transitions but also by major variations in the climate. The objective of this study was to examine differences in diet and mobility between distinct populations of the Peninsula and explore the possible relationship of diet, mobility, and culture with environmental variables and geographical settings. For this purpose, we obtained stable isotopic ratios of carbon and oxygen (δ^13^C and δ^18^O) from the enamel apatite of first upper incisors from 145 individuals at eight archeological sites that represent both Christian and Islamic communities and both rural and urban social settings. Results revealed a dietary difference between Christian and Islamic populations, observing a greater contribution of C_4_ plants, possibly sorghum, in the diet of the latter, especially in a rural setting. The disparity in oxygen isotopic ratios between populations from the North and South of the Peninsula is consistent with modern climatic differences between these regions. In this line, intraregional variability in oxygen isotopic ratios may hint at diachronic occupation phases under varying climatic conditions. The few isotopic outliers in our sample suggest overall low mobility levels.

## Introduction

The Middle Ages (8^th^-15^th^ centuries) in the Iberian Peninsula was an especially interesting period from multiple perspectives. Historically, it featured political occupation by Muslim groups and a long and complex period in which territory was recovered by different Christian kingdoms in the North of the Peninsula [1, 2]. From a cultural and social perspective, the Islamic arrival brought new habits and customs and novel techniques in craftwork and farming, with the introduction of new crops [3, 4]. This long chronological period also included climate changes, notably the Medieval Climate Anomaly (9^th^-11^th^ century) and the Little Ice Age (14^th^-15^th^ century), separated by a short transition phase in the 13^th^ century [5–7]. However, there has been little investigation of the influence of these environmental events on cultural and social changes, especially in human remains, and further in-depth research is required.

One useful approach is offered by stable isotope analysis of the apatite in human tooth enamel [8]. Isotope analysis of bone can yield information on the last years of life of individuals [9], with results that vary according to the turnover rate of the bone under study. However, isotope analysis of tooth enamel is not affected by tissue remodeling [10–12] but rather reflects the diet consumed during its formation [13]. Hence, the results obtained refer to years during which the dental crown forms or to a specific time point during this process, depending on the sampling technique. These features make the isotopic signal dental enamel a useful marker of the ingested food and water, and ultimately the environmental variables, characterizing the place where one individual spent their childhood. There it follows the wide application of isotopic analyses of dental enamel in mobility research to pinpoint the presence, and possibly geographic origin of nonlocal individuals [14]. One potential limit to this approach is, however, the effect played by breastfeeding and weaning on the observed isotopic values [15].

The proportion of stable carbon isotopes (^13^C/^12^C) in tooth enamel apatite corresponds to the whole diet, including proteins, carbohydrates, and fats, whereas the proportion in collagen is largely related to its protein component [16–18]. Various authors have described the δ^13^C_en_ (VPDB) values associated with different types of diet [19–22], with values close to -14‰ being characteristic of environments with C_3_ plants, those closer to 0‰ reflecting C_4_ plant intake, generally in warmer climates [23], and values between -9.5 and -12.8‰ suggesting a mixed diet of C_3_ and C_4_ plants. Analysis of the proportion of ^18^O/^16^O isotopes is also used by archeologists to track mobility patterns [14, 24–26] because it varies geographically as a function of the sources of drinking water [26]. The isotopic proportion of body oxygen results from a complex development involving metabolic processes and specific factors such as body size [27–29]. The main contributor to the isotopic representation of oxygen in the body is drinking water [30, 31], and δ^18^O_dw_ values (dw = drinking water) are closely bound to this source [32, 33]. It should be noted that isotopic differences can be generated between local rainwater and water at a specific site due to its evaporation or travel through run-offs or streams, etc. [34, 35]. The isotopic proportion of drinking water is also influenced by climate factors [30, 36], and it therefore depends on multiple parameters, including latitude, altitude, season, precipitation level, temperature, and/or distance from the coast [25, 37].

Studies on the Iberian Peninsula during the Middle Ages have related tooth enamel isotopic values to diet and mobility patterns [24, 25, 38–41], but there has been less research on their association with the environment. These aspects are all considered in the present study, with a particular focus on climate change. Two starting hypotheses were formulated: δ^13^C_en_ values would reflect dietary differences between Christians (in the North of the Peninsula) and Muslims (in the South) that can be attributed not only to cultural variations but also to geographic and environmental factors, as suggested by other studies based on collagen analyses (e.g., [24, 42, 43]); and δ^18^O_dw_ values at each site would correspond to their geographic position and the chronology of their occupation and would also reflect climate changes experienced during the Middle Ages [6].

The main study objective was to determine possible differences in diet and climatic environment between Christian populations from the North of the Iberian Peninsula and Muslim populations from the South of the Peninsula. Secondary objectives were to detect possible territorial mobility patterns and to identify any dietary differences between social classes and between rural and urban dwellers.

## Material and methods

**Fig 1:**
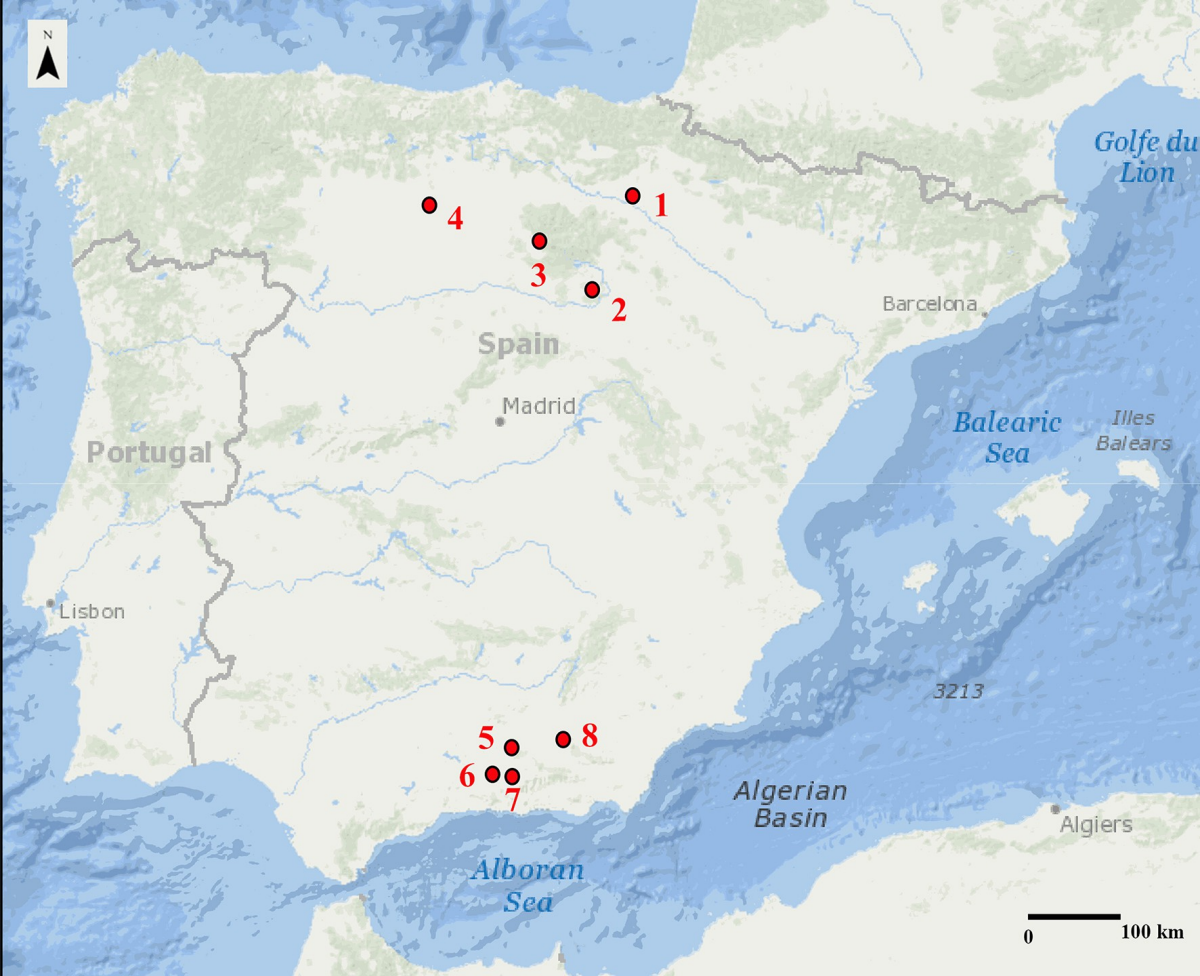
Geographical location of sites under study: 1.- Santa María de Tejuela; 2.- San Baudelio de Berlanga; 3.- El Castillo necropolis; 4.- La Olmeda; 5.- Sahl ben Malik cemetery; 6.- La Torrecilla; 7.- Talará cemetery; 8.- Mancoba. Map: https://apps.nationalmap.gov/viewer/ (11/04/2024).

The study included samples from 145 individuals at the Medieval sites depicted in Fig 1. Four sites are Christian rural villages from the North of the Iberian Peninsula, while four comprise two urban Muslim sites and two rural Muslim sites from the Southeast of the Peninsula. Table 1 exhibits their geographic coordinates, height above mean sea level (AMSL), and current climate according the Köppen-Geiger classification [44].

**Table 1:** Archaeological sites and their geographic coordinates, altitude, and climate.

Site	Latitude	Longitude	AMSL	Climate (Köppen-Geiger)
Tejuela	42° 45’ 32’’ N	3° 03’ 44’’ W	499 m	Cfb
San Baudelio	41° 25’ 06’’ N	2° 47’ 25’’ W	1049 m	Cfb
Palacios	41° 57’ 48’’ N	3° 07’ 34’’ W	1068 m	Csb
Olmeda	42°, 28’, 54’’ N	4°,44’, 11’’ W	890 m	Csb
Granada	37° 11’ 03’’ N	3° 36’ 00’’ W	694 m	Csa
Torrecilla	36° 58’ 22’’ N	3° 52’ 39’’ W	830 m	Csa
Talará	36° 57’ 00’’ N	3° 32’ 39’’ W	743 m	Csa
Baza	37° 29’ 03" N	2° 46’ 41" W	844 m	BSk

AMSL: Height above mean sea level; Cfb: temperate humid climate with mild summers; Csb: temperate climate with dry and mild summers; Csa: temperate climate with dry and warm summers; BSk: semi-arid temperate/cold climate. All climate data were obtained from https://es.climate-data.org

### Santa María de Tejuela (Bozoó, Burgos)

The remains of 182 individuals have been recovered from this cemetery (henceforth Tejuela) of a small rural community beside the Ebro River [45, 46]. Absolute dating values range from the late 8^th^ century to the beginning of the 11^th^ century, and it was mainly used between the mid-9^th^ and late 10^th^ centuries [47], i.e., during the Medieval Climate Anomaly [6, 7]. Samples were taken from 10 males and 10 females whose sex and age were previously estimated [47, 48].

### San Baudelio de Berlanga (Berlanga de Duero, Soria)

At least 57 individuals have been recovered from the small cemetery of San Baudelio hermitage used alongside the 11^th^ century [45, 49]. This small farm was mainly inhabited by stockbreeders with very hard conditions of life, supplied with water from a natural spring and the nearby Escalote River [50]. Individualized skeletons were dated from the 11^th^ to mid-13^th^ century, i.e., between the Medieval Climate Anomaly and the period of transition to the Little Ice Age [6, 51]. Samples were taken from 13 males and 7 females [51].

### El Castillo necropolis (Palacios de la Sierra, Burgos)

Over 100 very poorly preserved skeletons have been recovered from the cemetery on a hill beside the Arlanza River (henceforth Palacios) [45, 52, 53]. The local economy, determined by the high altitude and climate, centered on stockbreeding and the exploitation of forest resources [54–56]. According to epigraphs on the tombs and absolute dating values [53, 57], the cemetery was occupied from the 9^th^ to 13^th^ centuries, i.e., between the Medieval Climate Anomaly and the period of transition to the Little Ice Age [5–7]. Samples were taken from 6 females [48].

### La Olmeda (Pedrosa de la Vega, Palencia)

At least 239 individuals have been recovered from this early Medieval cemetery (henceforth Olmeda) on the right bank of the Carrión River, occupied from the 7^th^ to 13^th^ centuries [58, 59]. Despite the importance of the osteological collection, only a few studies have been published, largely focused on the paleopathology of the skulls [60]. Samples were taken from 9 males and 10 females, mainly from the Medieval Climate Anomaly [5, 7, 61].

### Sahl-ben-Malik cemetery (Cuesta del Hospicio sector, Granada)

Henceforth Granada cemetery, was the most important in the city of Granada during Medieval times [62]. It was occupied from the 11^th^ to 15^th^ century [63, 64], and most tombs date from the transition to or during the Little Ice Age [5, 6]. Samples were taken from 18 males and 12 females found under the present-day street called Cuesta del Hospicio. During Medieval times, Granada was supplied with water by the Darro and Genil Rivers and by the Aynadamar *acequia* from the *Fuente Grande* spring in nearby Alfacar [65, 66].

### La Torrecilla (Arenas del Rey, Granada)

At least 152 individuals have been recovered in varied states of preservation from this cemetery (henceforth Torrecilla) of a small rural settlement on the Cacín River plain [67, 68]. According to radiocarbon dating values, most tombs are from the 13^th^ to 15^th^ centuries, i.e., the beginning of the Little Ice Age [6, 7]. Samples were taken from 11 males and 13 females [65, 69].

### Talará cemetery (Valle de Lecrín, Granada)

Over 80 individuals have been recovered from this cemetery (henceforth Talará), which belonged to a farmhouse or rural settlement comparable to Torrecilla [68]. It lies below Sierra Nevada in the Lecrín valley on a natural route between the Mediterranean coast and the city of Granada. The tombs date from the 14^th^ and 15^th^ centuries [70], i.e., the beginning of the Little Ice Age [5, 7]. Samples were taken from 10 males and 10 females.

### Mancoba (Baza, Granada)

Over 300 individuals have been recovered from this cemetery (henceforth Baza) in Baza, a city of special importance in the late Middle Ages due to its strategic location near the frontier with the Kingdom of Castile, its economic wealth, and its industrial production [71]. It was mainly supplied with water from the Guadiana Menor River [72]. According to radiocarbon dating, the cemetery was occupied in the 14^th^ and 15^th^ centuries. Samples were taken from 3 males and 4 females.

### Samples

All samples studied (n = 145) were from upper first incisors unaffected by trauma, caries, or wear extracted from individuals aged over 20 years. The distal half of the crown analyzed corresponds to ages between 7 months and 4.5 years according to the atlas of AlQahtani et al., [73].

### Analytical procedures

Carbon (δ^13^C_en_VPDB) and oxygen (δ^18^O_c_ VPDB) isotope values were determined in the structural carbonate of the enamel of the 145 incisors; analyses were conducted in the Stable Isotope Biogeochemistry Laboratory of the *Instituto Andaluz de Ciencias de la Tierra* (CSIC, Granada, Spain) using standard protocols [74, 75]. After scanning the teeth with an Artec Micro scanner and cleaning the dental surface to eliminate possible contaminants, the enamel was mechanically extracted with a microdrill and then pulverized. Next, ~10 mg of this powder was dissolved and processed, using chemical reagents to eliminate organic matter (1 mL 1.75% sodium hypochlorite for 45 min) and secondary carbonates (1 mL 0.1 M acetic acid for 15 min). Samples were subsequently lyophilized, treated with helium to eliminate atmospheric gases, and injected with 100% phosphoric acid at 72°C for ~ 2.5 h. Samples (burned to form CO_2_) were placed in a gas chromatographer (Finnigan Gas Bench II) and then in a Thermo Finnigan DELTA plus XP IRMS to measure isotope ratios, using two international standards (NBS18 and NBS19) and one internal standard (Cavendish Marble). All results were referred to the standard Vienna PeeDee Belemnite (VPDB) and expressed in delta (δ) notation as ‰: δ^18^O = ((^18^O/^16^O sample) / (^18^O/^16^O standard))– 1) * 1000 [8].

### Statistical procedures

IBM SPSS 22 was used for statistical analyses. The Shapiro-Wilk test was applied to test distribution normality. The mean absolute deviation from the median multiplied by three (±3MAD_norm_) was used as a robust measure of scale for small sample sizes and non-normal distributions to detect outliers, considered appropriate for most biological data [14, 76]. We also considered ±2 standard deviations from the mean. The non-parametric Mann-Whitney test was performed to evaluate between-group and between-sex differences, setting statistical significance at 0.05.

Enamel δ^18^O_c_ (VPDB) values were transformed into phosphate (δ^18^O_p_VSMOW) and drinking water (δ^18^O_dw_VSMOW) values for comparisons with δ^18^O_tsw (_VSMOW) results for terrestrial surface water sources. The best procedure for this data transformation remains under debate [10, 30, 77], and we used three widely applied linear equations [78–81, among others]:

δ^18^O_c_ (VSMOW) = (1.03091 * δ^18^O_c_ (VPDB)) + 30.91 [82]

δ^18^O_p_ (VSMOW) = (1.122 * δ^18^O_c_ (VSMOW))– 9.6849 [10]

δ^18^O_p_ (VSMOW) = (0.78 * δ^18^O_dw_ (VSMOW)) + 22.70 [33]

δ^18^O_c_ (VSMOW) values were transformed into δ^18^O_p_ (VSMOW) values by using a combination of Iacumin et al. [83] and Metcalfe et al. [84] linear equations for arid climates [10], which provide a satisfactory fit with the δ^18^O_tsw_ (VSMOW) values from the study areas.

## Results

**Fig 2:**
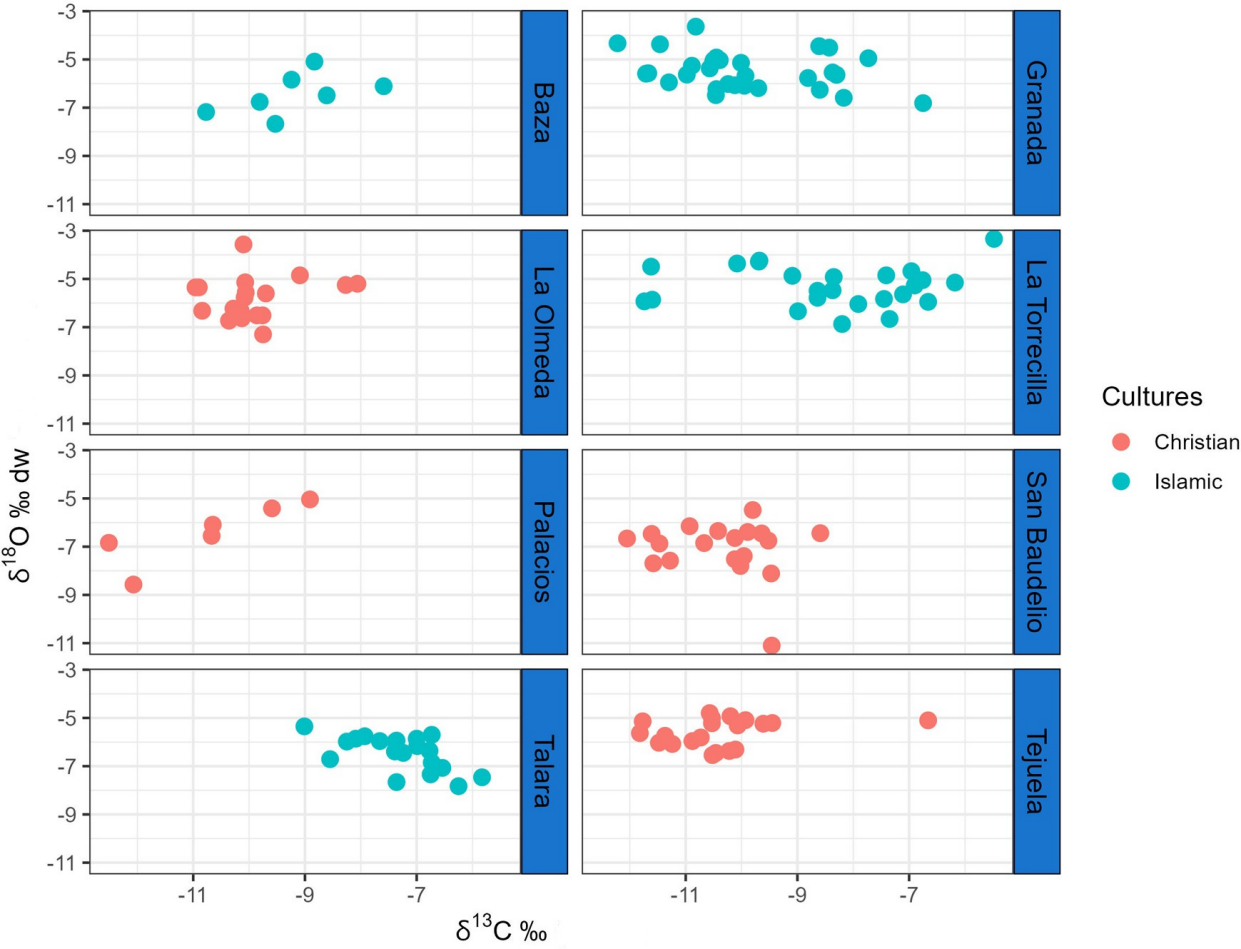
Individual δ^13^C_en_ (VPDB) and δ^18^O_dw_ (VSMOW) values by site and cultural group.

**Fig 3:**
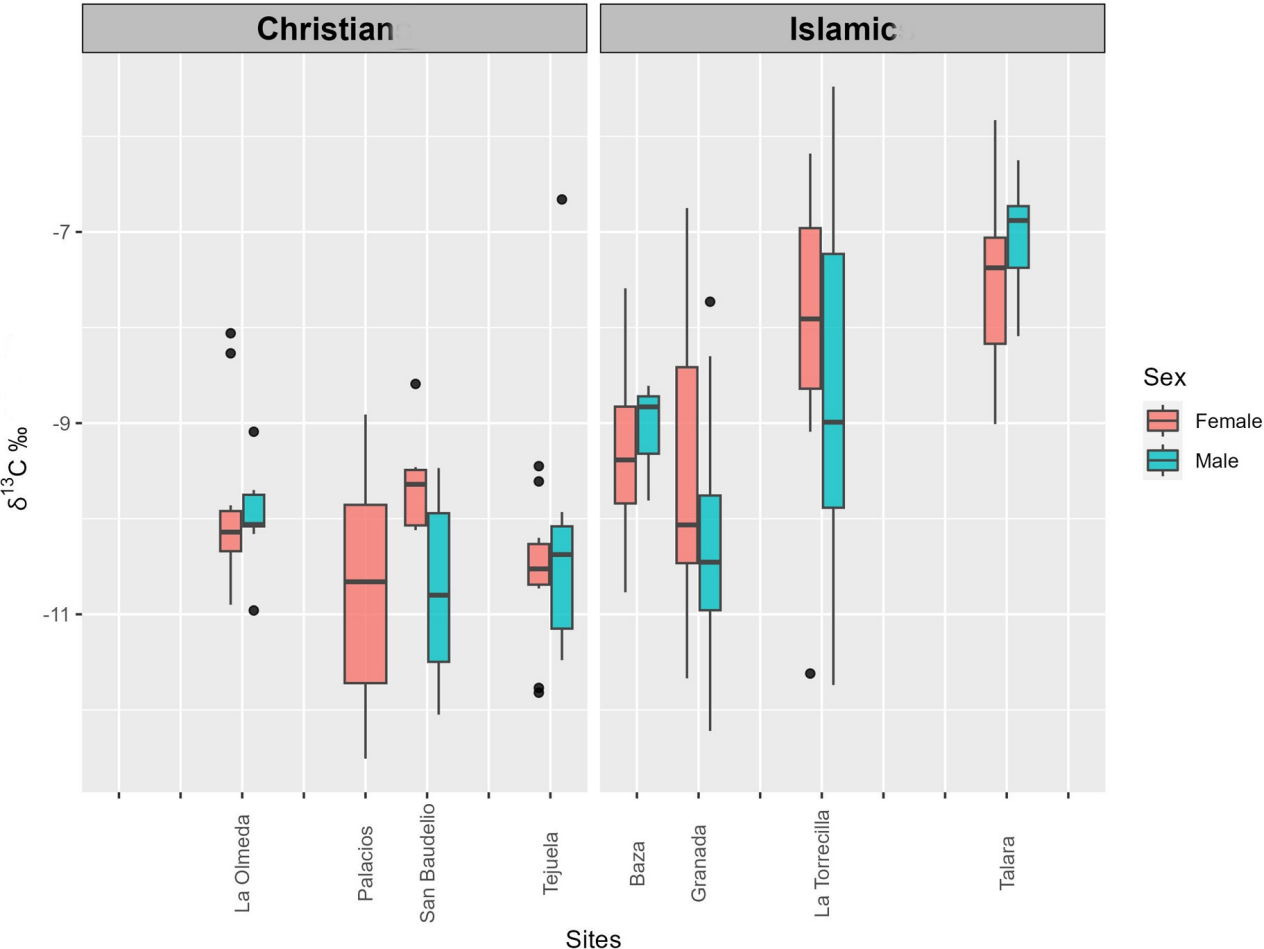
Box plot of δ^13^C_en_ (VPDB) values at sites by cultural group and sex.

**Fig 4:**
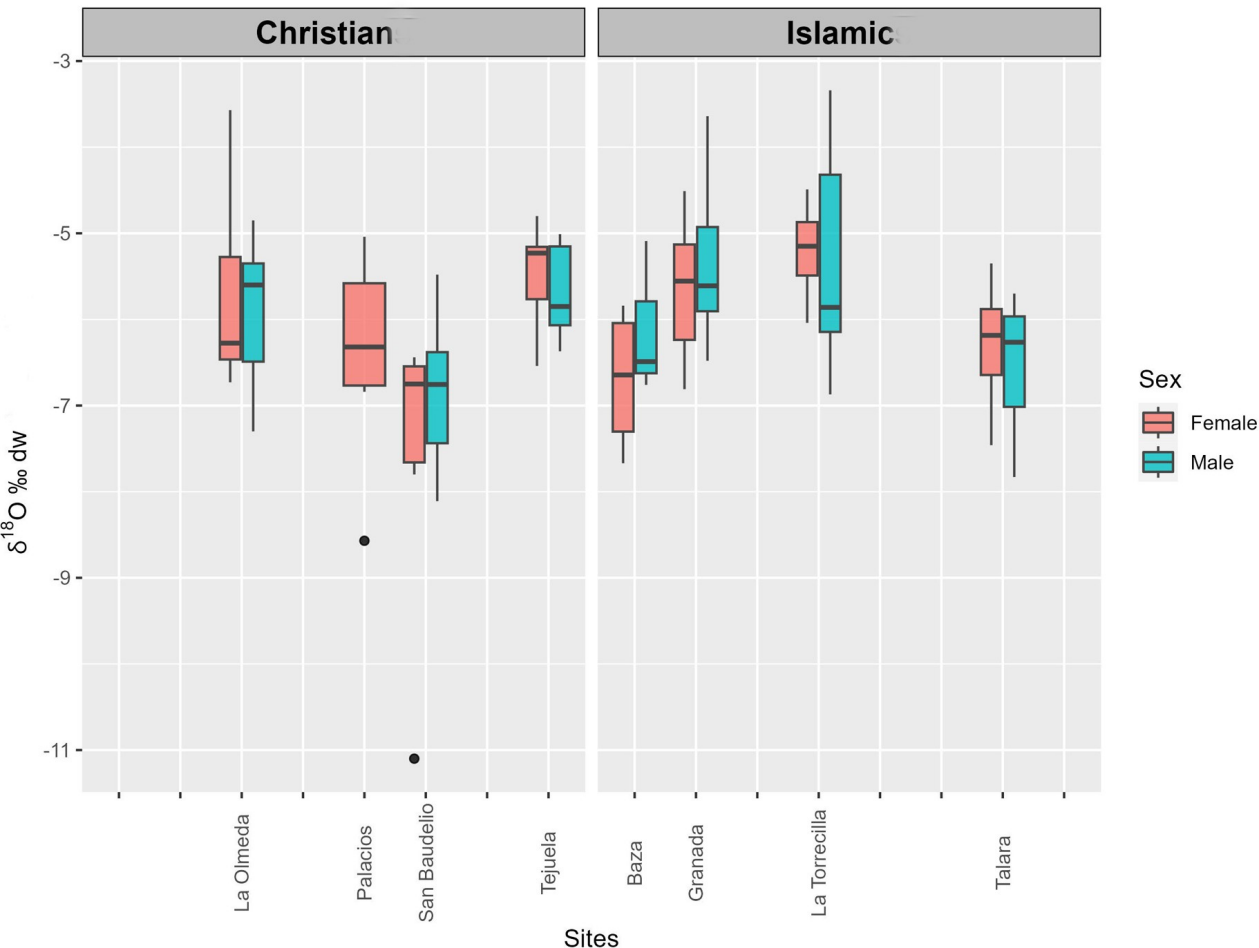
Box plot of δ^18^O_dw_ (VSMOW) values at sites by cultural group and sex.

S1 Table lists the δ^13^C_en_ (VPDB), δ^18^O_c_ (VPDB), and δ^18^O_dw_ (VSMOW) values obtained for each sample and reports the sex of the individual. Table 2 exhibits mean δ^13^C_en_, δ^18^O_c_ and δ^18^O_dw_ values by site and sex. Fig 2 depicts δ^13^C_en_ and δ^18^O_dw_ values by site and cultural group. Figs 3 and 4 depict the distribution of δ^13^C_en_ and δ^18^O_dw_ values by sex and cultural group.

**Table 2:** Mean δ^13^C_en_, δ^18^O_c_, and δ^18^O_dw_ values by site for the whole sample and by sex.

**δ**^**13**^**C**_**en**_ **‰ (VPDB)**									
Site	Total			Males			Females		
	n	Min-max	Mean ± σ	n	Min-Max	Mean ± σ	N	Min-Max	Mean ± σ
TE	20	-11.8–6.7	-10.4 ± 1.1	10	-11.5–6.7	-10.2 ± 1.4	10	-11.8–9.4	-10.5 ± 0.8
SB	19	-12.0–8.6	-10.3 ± 0.9	12	-12.0–9.5	-10.8 ± 0.8	7	-10.1–8.6	-9.6 ± 0.5
OL	19	-11.0–8.1	-9.9 ± 0.8	9	-11.0–9.1	-9.9 ± 0.5	10	-10.9–8.1	-9.9 ± 1.0
PA	6	-12.5–8.9	-10.7 ± 1.4	-	-	-	6	-12.5–8.9	-10.7 ± 1.4
Total North	64	-12.5–6.6	-10.3 ± 1.0	31	-12.0–6.7	-10.4 ± 1.0	33	-12.5–8.1	-10.2 ± 1.0
GR	30	-12.2–6.7	-9.9 ± 1.3	18	-12.2–7.7	-10.2 ± 1.2	12	-11.7–6.7	-9.5 ± 1.4
TO	24	-11.7–5.5	-8.4 ± 1.7	11	-11.7–5.5	-8.9 ± 2.0	13	-11.6–6.2	-8.0 ± 1.4
TA	20	-9.0–5.8	-7.3 + 0.8	10	-8.1–6.2	-7.0 ± 0.6	10	-9.0–5.8	-7.5 ± 0.9
BA	7	-10.8–7.6	-9.2 ± 1.0	3	-9.8–8.6	-9.1 ± -	4	-10.8–7.6	-9.3 ± 1.3
Total South	81	-12.2–5.5	-8.8 ± 1.7	42	-12.2–5.5	-9.0 ± 1.8	39	-11.7–5.8	-8.5 ± 1.5
**δ**^**18**^**O**_**c**_ **‰ (VPDB)**									
Site	Total			Males			Females		
	n	Min-max	Mean ± σ	n	Min-Max	Mean ± σ	N	Min-Max	Mean ± σ
TE	20	-4.3–3.1	-3.6 ± 0.4	10	-4.2–3.2	-3.7 ± 0.4	10	-4.3–3.1	-3.6 ± 0.4
SB	19	-7.7–3.6	-4.7 ± 0.8	12	-5.5–3.7	-4.5 ± 0.5	7	-7.7–4.3	-5.1 ± 1.2
OL	19	-4.9–2.2	-3.8 ± 0.6	9	-4.9–3.1	-3.8 ± 0.6	10	-4.5–2.2	-3.8 ± 0.7
PA	6	-5.8–3.2	-4.2 ± 0.9	-	-	-	6	-5.8–3.2	-4.2 ± 0.9
Total North	64	-7.7–2.2	-4.1 ± 0.8	31	-5.5–3.1	-4.1 ± 0.6	33	-7.7–2.2	-4.1 ± 1.0
GR	30	-4.8–2.2	-3.5 ± 0.6	18	-4.1–2.2	-3.4 ± 0.5	12	-4.8–2.9	-3.7 ± 0.6
TO	24	-4.6–2.0	-3.4 ± 0.6	11	-4.6–2.0	-3.5 ± 0.8	13	-3.8–2.8	-3.3 ± 0.3
TA	20	-5.3–3.5	-4.3 ± 0.5	10	-5.3–3.7	-4.3 ± 0.6	10	-5.0–3.5	-4.2 ± 0.5
BA	7	-5.2–3.3	-4.3 ± 0.5	3	-4.5–3.3	-4.1 ± -	4	-5.2–3.8	-4.5 ± 0.6
Total South	81	-5.3–2.0	-3.7 ± 0.7	42	-5.3–2.0	-3.7 ± 0.7	39	-5.2–2.8	-3.8 ± 0.6
**δ** ^**18**^ **O**_**dw**_ **‰ (VSMOW)**									
Site	Total			Males			Females		
	n	Min-max	Mean ± σ	n	Min-Max	Mean ± σ	N	Min-Max	Mean ± σ
TE	20	-6.5–4.8	-5.6 ± 0.6	10	-6.4–5.0	-5.7 ± 0.5	10	-6.5–4.8	-5.5 ± 0.6
SB	19	-11.1–5.5	-7.1 ± 1.2	12	-8.1–5.5	-6.8 ± 0.7	7	-11.1–6.4	-7.5 ± 1.7
OL	19	-7.3–3.6	-5.8 ± 0.9	9	-7.3–4.8	-5.8 ± 0.8	10	-6.7–3.6	-5.8 ± 1.0
PA	6	-8.6–5.0	-6.4 ± 1.2	-	-	-	6	-8.6–5.0	-6.4 ± 1.2
Total North	64	-1.1–3.6	-6.2 ± 1.1	31	-8.1–4.8	-6.2 ± 0.9	33	-11.1–3.6	-6.2 ± 1.3
GR	30	-6.8–3.6	-5.5 ± 0.7	18	-6.5–3.6	-5.3 ± 0.8	12	-6.8–4.5	-5.7 ± 0.7
TO	24	-6.9–3.3	-5.3 ± 0.8	11	-6.9–3.3	-5.4 ± 1.1	13	-6.0–4.5	-5.2 ± 0.5
TA	20	-7.8–5.3	-6.4 ± 0.7	10	-7.8–5.7	-6.6 ± 0.8	10	-7.5–5.3	-6.3 ± 0.7
BA	7	-7.7 .5.1	-6.4 ± 0.9	3	-6.8–5.1	-6.1 ± -	4	-7.7–5.8	-6.7 ± 0.9
Total South	81	-7.8–3.3	-5.7 ± 0.9	42	-7.8–3.3	-5.7 ± 1.0	39	-7.7–4.5	-5.8 ± 0.8

TE: Tejuela; SB: San Baudelio; OL: La Olmeda; PA: Palacios; GR: Granada; TO: La Torrecilla; TA: Talará; BA: Baza

Regarding δ^13^C_en_ values, only one male at Tejuela (TE 26) and two females at Olmeda (OL 11 and OL 112) are outliers by ±3MAD_norm_, with one male at Torrecilla (TO 12) being very close to the ±3MAD_norm_ cutoff. The same four individuals are outliers when ±2σ is applied (Fig 3). In relation to δ^18^O_dw_ values, only one female at San Baudelio (SB 14) is an outlier by ±3MAD_norm_, although a female at Olmeda (OL 167) and a male at Torrecilla (TO 28) are outliers by ± 2σ, and a male at Torrecilla (TO 2) and a male at Talará (TA 19) are at the cutoff point (Fig 4).

The distribution of δ^13^C_en_ values did not differ among the four Christian sites in the Northern Peninsular or between the males and females at these sites, with the exception of a less negative mean value for females *versus* males at San Baudelio (p = 0.02). However, their distribution significantly differed (p<0.001) among the four Islamic sites in the South, with markedly less negative values at the rural (Torrecilla and Talará) *versus* urban (Granada and Baza) sites, while no significant between-sex difference was found at any site or between rural and urban sites. δ^13^C_en_ values significantly differed between the Christian and Islamic sites (p<0.001), observing a considerably less negative values in the latter.

Among the Christian sites, significantly lower mean δ^18^O_c_ and δ^18^O_dw_ values (p<0.001) were recorded at San Baudelio than at the remaining sites except for Palacios, with no differences among the other sites. No between-sex difference in these values was observed at any site. Among Islamic sites, significantly lower mean δ^18^O_c_ and δ^18^O_dw_ values (p<0.001) were observed at Baza and Talará than at Granada and Torrecilla, with no difference between Baza and Talará (p = 0.85) or between Granada and Torrecilla (p = 0.41). No between-sex difference in these values was observed at any site. Both δ^18^O_c_ and δ^18^O_dw_ values were significantly lower (p<0.02) at the Christian sites in the North than at the Islamic sites in the South.

## Discussion

### Dietary patterns

The δ^13^C_en_ values suggest that the diet of the individuals from Tejuela incorporated a large portion of C_3_ plants, at least during their early childhood. However, the intake of some C_4_ type plants cannot be ruled out, especially common millet (*Panicum miliaceum*) and foxtail millet (*Setaria italica*), cultivated in the Iberian Peninsula since the Early Iron Age [85]. The diet of the outlier (TE 26) indicates a major consumption of C_4_ type plants. These data are not at odds with the average δ^13^C in bone collagen samples of -18.6 ± 0.6‰ (VPDB) previously calculated for the same cemetery [57], which pointed to a substantial dietary contribution of C_3_ plants while not excluding a possibly lesser consumption of C_4_ species. Similar values are encountered at San Baudelio, although the sexes significantly differed at this site, where the females showed a higher consumption of C_4_ plants. A similar difference was observed in δ^13^C_col_ (bone collagen) values, which were a mean of -18.2±0.4‰ (VPDB) in the females [51], attributable to their distinct geographic origin. The δ^13^C_en_ values at Olmeda also reveal a mixed diet with a predominance of C_3_ plants but a higher consumption of C_4_ plants than at Tejuela or San Baudelio. The fact that these individuals lived during the Medieval Climate Anomaly may explain their greater intake of C_4_ plants, which are more characteristic of warmer environments, with the values simply corresponding to these types of climatic conditions [86]. The particularly high consumption of C_4_ plants by the two outlier females (OL 11, OL 112) at La Olmeda may indicate their early childhood in a different warmer location. Finally, δ^13^C_en_ values for the six females at Palacios suggest that they shared a C_3_-based diet with the possible occasional intake of C_4_ plants. The mean δ^13^C_col_ (bone collagen) value of -19.4 ± 0.5‰ (VPDB) in Palacios [57] is consistent with the intake of C_3_ plants. Interestingly, the lowest mean values were found in the males at San Baudelio and females at Palacios (Table 3), which are at the highest altitudes and would therefore have experienced colder climates.

**Table 3:** Isotopic values of δ^18^O_dw_ (VSMOW) in local water from different Spanish stations.

Station	n	Min	Max	Mean δ^18^O‰ (VSMOW) ± σ	Reference
Burgos (Villafria)	12	-11.9	-2.8	-7.7 ± 3.1 *	[101]
Soria	12	-11.7	1.9	-8.0 ± 3.7 *	[101]
Leon	16	-9.7	-6.3	-8.4 ± 1.0 *	[102]
Valladolid	16	-9.1	-7.0	-8.0 ± 0.7 *	[102]
Granada	44	-11.4	-2.6	-6.9 ± 2.5 *	[103]
Granada av				-9.4	[104]
Linares	7	-7.9	-3.0	-5.5 ± 1.7 *	[105]
Baza av				-9.08	[104]
Berchules av		-10.73	-8.26		[106]

*: standard deviation calculated by the authors; av: absolute value

Among the Islamic sites in the South of the Peninsula, the largest number of individuals are from the city of Granada. Their δ^13^C_en_ values are highly variable and indicate a mixed diet of both C_3_ and C_4_ plants, with a predominance of the former, as also observed in Baza, the other Islamic urban center studied. These results are consistent with the mean δ^13^C value of -17.1±1.2‰ (VPDB) previously reported in collagen from Granada [87], which also suggests a mixed diet with a major contribution of C_3_ plants. Clear differences were found between rural and urban Islamic sites. Samples from rural Torrecilla reveal a wide inter-individual variability, with an important intake of C_4_ plants that was very high in some individuals (e.g., TO 28). A lesser variability in values was observed at the other rural site, Talará, which showed an elevated consumption of C_4_ plants. Mean δ^13^C values in collagen are -16.0 ± 1.5‰ (VPDB) at Torrecilla and -15.3 ± 0.8‰ (VPDB) at Talará [69], in full agreement with the results obtained in enamel apatite. The similarity of results with the isotopic values from adult collagen in the studied collections suggests that, throughout the weaning process, the children received supplementary food incorporating the same or possibly similar plant resources as the rest of the community. The variation between the North and South of the Peninsula can be attributed to differences in climate [88, 89] and diet.

When the Arabs arrived in the Iberian Peninsula (8^th^ century), they engaged in a complex process of introduction, reimplantation, and diffusion of certain plants, some of which were acclimatized in the botanic gardens of the royal courts [3]. Citruses, rice, and the C_4_ plants sugarcane and sorghum subsequently reached kingdoms in the North of the Peninsula [90]. Sugarcane is solely a carbohydrate and contains no amino acids, hampering its detection by isotopic analysis, although ~3% of natural sugarcane juice and molasses is protein [91, 92], which would enrich its δ^13^C_en_ value. Sugar was used in pharmacy and cooking but was always a luxury item reserved for special occasions, mainly among the wealthier classes [93, 94]. However, its consumption increased in the 14^th^-15^th^ century during Nasrid times, and the only site of sugarcane cultivation during the late Middle Ages was on the Mediterranean coast of Granada province [95]. Indeed, the 14^th^ century Granada writer Ibn al-Jatib recommended the use of sugarcane and crystalized sugar to calm children during weaning (in the second year of life) [93], and the individuals in this study may therefore have consumed sugar during their childhood [96], although it is not possible in our study to further precise the age of weaning process.

The other C_4_ type vegetable, sorghum, must have adapted very well to conditions in Granada, whose climate and altitude do not favor the cultivation of wheat, the cereal in greatest demand [97]. Wheat was mainly imported from North Africa and reserved for wealthier families [90, 94, 98]. Ibn-al Jatib and other contemporary writers such as Abū l-Jayr and Ibn Razin al-Tuŷībī underscored the nutritional value of wheat, describing sorghum as a cereal consumed by less favored social classes and rural populations and in times of shortage [90, 94, 98]. This would explain the greater consumption of sorghum at rural sites such as Torrecilla and Talará, while social differences would account for the variability of isotopic values in urban areas.

### Environment

Although our complete dataset features 145 individuals, the study is limited by the very small sample size for some sites (e.g., Palacios or Baza), where it was not possible to detect any significant between-sex differences. In addition, the conversion of ^18^O_c_ (VPDB) to δ^18^O_dw_ (VSMOW) is affected by a high predictive error [10, 14, 30, 77, 99], which is why Table 2 displays both values for each individual. Furthermore, the comparisons are based on isotopic oxygen data for water that does not always derive from the precise location of the site. The characteristics of current water may vary from those during the investigated period, and there may be differences between the origin of the water (rainfall or rivers) and the water consumed at the site.

Among the sites in the Northern Peninsular, the highest mean δ^18^O_c_ and δ^18^O_dw_ values were observed at Tejuela, likely because it has lowest altitude above sea level (Table 1) and was occupied during the Medieval Climate Anomaly. Similar values are recorded at the site of La Olmeda, which is at a greater height above sea level and has a drier climate but was also occupied during the Medieval Climate Anomaly [5, 6]. No between-sex differences in these values were observed at either site. The lowest mean δ^18^O_c_ and δ^18^O_dw_ values were found at San Baudelio and were similar to those observed at Palacios, with both sites being located at the highest altitudes (Table 1). A much lower value is shown by one female (SB 14) at San Baudelio. Both San Baudelio and Palacios were occupied during the phase of transition to the Little Ice Age [6, 7]. In the present day, all four sites have similar climate, with mild summers, although Tejuela and San Baudelio are wetter (Table 1). There are also no differences in current rainwater δ^18^O_dw_ values among these areas (Table 3), all in a similar geographic environment on the North sub-plateau, a large sedimentary basin surrounded by high mountain ranges where relatively flat lands are crossed by fluvial valleys, with a mean height of 750 m a.s.l., mild and dry summers, and cold winters [100]. Hence, differences among the Northern sites may be attributable to distinct climatic conditions at the time of their occupation rather than to climate differences within the region.

Differences in mean values between the Christian sites in the North of the Iberian Peninsula and the Islamic sites in the South can be explained by their distinct ecosystems [88, 89]. The area of Granada is characterized by a richness and variety of biotopes favored by the presence of the mountain range of Sierra Nevada, which acts as a geological and geographic barrier, by its proximity to the sea, and by the presence of meadows and high plateaus [107]. This would account for different climatic conditions in Baza (Table 1) and the highly varied rainwater δ^18^O_dw_ values in the present day. Mean findings in Granada and Torrecilla are similar to current values in Granada city and Linares (Jaen). Baza is currently the coldest site, with a mean minimum temperature in January of -0° C [108], which may explain the more negative mean oxygen finding. However, a similar mean value was recorded at Talará, although the climate at this site is the same as that of Granada and Torrecilla. This discrepancy may result from its localization in the Valley of Lecrín at the foot of Sierra Nevada, given that water supplied by rivers from the mountains or by springs fed by melted ice would have lower oxygen isotopic values [109]. In this way, rainwater oxygen values from the nearby village of Bérchules, also at the foot of Sierra Nevada, are lower than those of Granada and Linares and similar to those in Baza (Table 3). Besides the geographic and climatic differences among these sites, both Granada and Torrecilla included individuals that lived in the phase of transition to the Little Ice Age and all individuals at Baza and Talará lived during this period [6, 7].

### Mobility

Some individuals are outliers by both ±3MAD_norm_ and ± 2 standard deviations from the mean, indicating a possible non-local origin. We highlight the δ^18^O_dw_ of the female (SB 14) at San Baudelio, where a previous study [51] found that the carbon ratios in bone collagen significantly differed between females and males, suggesting the distinct origin of the former. No female at this site had reached the age of 30 years, and the authors proposed that the results would be attributable to the diet consumed before they left their place of origin to marry elsewhere. San Baudelio was a small farm that only had space for around two or three families [50], and it would have been necessary to bring in females to avoid consanguinity. This proposal is supported by the present δ^13^C_en_ data, which indicate that the diet of the males and females differed during their first years of life. In fact, carbon isotope values were higher in the females than in the males, which may suggest that they came from a warmer environment. On the other hand, the similar oxygen isotope values between the females and males suggest that their place of origin would not have been very distant and may have had a similar climate but at a lower altitude, although the much lower oxygen isotope value in the female outlier (SB 14) indicates an origin in a much colder climate. Two females at La Olmeda (OL11 and OL112) are outliers by δ^13^C_en_ and another (OL 167) is an outlier by δ^18^O_dw_, and they likely came from a warmer place, especially OL 167. This may suggest an exogamic pairing, a common practice in Medieval Castile [110]. However, although C_4_ plants are more common in warmer environments, supporting this proposition, the results for OL11 and OL112 may also have a socioeconomic explanation, with the consumption of less valued C_4_ plants [97] pointing to a lower social class, in line with other medieval findings in the Iberian Peninsula [42]. Among males, the diet of one adult male (TE 26) during childhood markedly differed from that of other individuals at Tejuela, whose demography indicates the recent foundation of this village [111]. This result again raises the possibility that his early childhood was spent in a warmer environment but may also reflect a lower social status. Among the Islamic sites in the South of the Peninsula, two males (TO 2 and TA 19) appear to have spent their childhood in a colder environment and may possibly have moved to the last Islamic territory remaining after the southward advance of the Christian Monarchs, i.e., the Nasrid Kingdom of Granada [112]. In contrast, one male at Torrecilla (TO 28) appears to have come from a much warmer place and may possibly have spent his childhood in Northern Africa, with this type of migration being documented in historical sources [113]. It is worth mentioning that the breastfeeding period and the weaning process may have played some influence on the oxygen isotopic ratios of the analyzed individuals [15]. Body water, and therefore breast milk, features higher δ^18^O values than drinking water [114]. When considering data from different teeth featuring different forming periods, this leads to a descending curve in the diachronic trajectory of oxygen isotopic ratios (from enriched to the level of the local drinking water [115–117]. It is clear that these patterns, if not taken into account, may mimic (or mask) isotopic differences related to environmental variables. In our case, this potential bias cannot be clarified due to the applied analytical approach but it needs to be taken into account.

## Conclusions

This study provides insights into dietary differences between Medieval populations from the Christian North and Islamic South of the Iberian Peninsula. There was a greater intake of C_4_ plants by the Muslims, likely attributable to their introduction of sugarcane and sorghum. This cereal is typical of warm climates, and its quality is considered inferior to that of wheat. C_4_ plant consumption was also higher in rural *versus* urban Islamic sites. Oxygen isotope values from enamel apatite differ between populations in the North and South of the Peninsula, consistent with the differences in their climates. Isotopic variability within each region can be attributed to diachronic occupation phases under varying climatic conditions. The analyses identified a few individuals who were possibly nonlocal, including females who may have arrived from elsewhere for the purpose of marriage.

## Supporting information

S1 Table Individual δ^13^C_en_, δ^18^O_c_, and δ^18^O_dw_ values. TE: Tejuela; SBB: San Baudelio; OL: La Olmeda; PA: Palacios; GR: Granada; TO: La Torrecilla; TA: Talará; BA: Baza. UI1: first upper incisor.
(DOCX)
