# Supplementary material for: North and South in Medieval Iberia: A historical and environmental estimate through isotopic analyses
Source: PLoS One. 2024 Jun 5;19(6):e0304313. doi: 10.1371/journal.pone.0304313 (PMC11152309; doi:10.1371/journal.pone.0304313)
Supplement: S1 Table — TE: Tejuela; SBB: San Baudelio; OL: La Olmeda; PA: Palacios; GR: Granada; TO: La Torrecilla; TA: Talará; BA: Baza. UI1: first upper incisor. (DOCX) [file pone.0304313.s001.docx]

| **Site** | **Individual** | **Sex** | **Tooth** | **δ^13^C‰ (VPDB)** | **δ^18^O_c_‰ (VPDB)** | **δ^18^O_dw_‰ (VSMOW)** |
| --- | --- | --- | --- | --- | --- | --- |
| TE | 25 | female | UI1 | -9.6 | -3.4 | -5.2 |
| TE | 26 | male | UI1 | -6.7 | -3.3 | -5.1 |
| TE | 33 | female | UI1 | -10.5 | -4.3 | -6.5 |
| TE | 34 | male | UI1 | -10.2 | -4.2 | -6.4 |
| TE | 35 | male | UI1 | -11.5 | -4.0 | -6,0 |
| TE | 36 | female | UI1 | -9.5 | -3.4 | -5.2 |
| TE | 50 | male | UI1 | -10.1 | -4.2 | -6.3 |
| TE | 54 | female | UI1 | -10.2 | -3.2 | -4.9 |
| TE | 82 | female | UI1 | -10.5 | -4.3 | -6.5 |
| TE | 83 | male | UI1 | -10.9 | -3.9 | -6.0 |
| TE | 124 | male | UI1 | -9.9 | -3.3 | -5,0 |
| TE | 146 | male | UI1 | -11.4 | -3.8 | -5.7 |
| TE | 148 | female | UI1 | -10.6 | -3.1 | -4.8 |
| TE | 151 | female | UI1 | -10.7 | -3.8 | -5.8 |
| TE | 180 | female | UI1 | -10.5 | -3.4 | -5.2 |
| TE | 252 | female | UI1 | -11.8 | -3.3 | -5.1 |
| TE | 259 | female | UI1 | -11.8 | -3.7 | -5.6 |
| TE | 263 | male | UI1 | -11.2 | -4.0 | -6.1 |
| TE | 265 | male | UI1 | -10.1 | -3.4 | -5.3 |
| TE | 266 | male | UI1 | -10.5 | -3.2 | -5.0 |
| SBB | 1 | female | UI1 | -10.0 | -5.3 | -7.8 |
| SBB | 2 | male | UI1 | -10.4 | -4.2 | -6.4 |
| SBB | 4 | female | UI1 | -9.5 | -4.5 | -6.8 |
| SBB | 5 | female | UI1 | -9.6 | -4.3 | -6.5 |
| SBB | 6 | female | UI1 | -10.1 | -4.4 | -6.6 |
| SBB | 7 | male | UI1 | -9.8 | -3.6 | -5.5 |
| SBB | 8 | female | UI1 | -10.1 | -5.1 | -7.5 |
| SBB | 9 | male | UI1 | -9,5 | -4.9 | -7.4 |
| SBB | 10 | male | UI1 | -10.9 | -4.1 | -6.2 |
| SBB | 11 | male | UI1 | -11.3 | -5.1 | -7.6 |
| SBB | 12 | male | UI1 | -11.6 | -5.2 | -7.7 |
| SBB | 13 | male | UI1 | -12,0 | -4.4 | -6.7 |
| SBB | 14 | female | UI1 | -9.5 | -7.7 | -11.1 |
| SBB | 15 | male | UI1 | -11.6 | -4.3 | -6.5 |
| SBB | 16 | male | UI1 | -10.7 | -4.6 | -6.9 |
| SBB | 17 | male | UI1 | -11.5 | -4.8 | -6.9 |
| SBB | 18 | female | UI1 | -8.6 | -4.3 | -6.4 |
| SBB | 19 | male | UI1 | -9.9 | -4.2 | -6.4 |
| SBB | 20 | male | UI1 | -9.5 | -5.5 | -8.1 |
| OL | 3 | female | UI1 | -9.9 | -4.3 | -6.5 |
| OL | 11 | female | UI1 | -8.1 | -3.4 | -5.2 |
| OL | 15 | male | UI1 | -10.1 | -3.8 | -5.8 |
| OL | 23 | female | UI1 | -10.1 | -4.4 | -6.6 |
| OL | 59 | female | UI1 | -10.8 | -4.2 | -6.3 |
| OL | 70 | female | UI1 | -10.4 | -4.5 | -6.7 |
| OL | 112 | female | UI1 | -8.3 | -3.4 | -5.3 |
| OL | 118 | male | UI1 | -10.1 | -3.6 | -5.6 |
| OL | 120 | male | UI1 | -9.8 | -4.3 | -6.5 |
| OL | 126 | male | UI1 | -11.0 | -3.5 | -5.4 |
| OL | 128 | female | UI1 | -10.3 | -4.1 | -6.2 |
| OL | 167 | female | UI1 | -10.1 | -2.2 | -3.6 |
| OL | 170 | male | UI1 | -10.1 | -3.3 | -5.1 |
| OL | 176 | female | UI1 | -10.1 | -4.2 | -6.3 |
| OL | 180 | male | UI1 | -10.2 | -4.3 | -6.5 |
| OL | 186 | male | UI1 | -9.7 | -3.7 | -5.6 |
| OL | 193 | male | UI1 | -9.8 | -4.9 | -7.3 |
| OL | 211 | female | UI1 | -10.9 | -3.5 | -5.4 |
| OL | 212 | male | UI1 | -9.1 | -3.1 | -4.9 |
| PA | 7 | female | UI1 | -8.9 | -3.2 | -5.0 |
| PA | 125 | female | UI1 | -10.7 | -4.0 | ¡-6.0 |
| PA | 248 | female | UI1 | -10.7 | -4.3 | -6.6 |
| PA | 249 | female | UI1 | -9.6 | -3.5 | -5.4 |
| PA | 445 | female | UI1 | -12.5 | -4.6 | -6.9 |
| PA | 499 | female | UI1 | -12.1 | -5.8 | -8.6 |
| GR | 1 | female | UI1 | -11.7 | -3.6 | -5.6 |
| GR | 2 | male | UI1 | -12.2 | -2.7 | -4.3 |
| GR | 3 | female | UI1 | -8.2 | -4.4 | -6.6 |
| GR | 5 | male | UI1 | -10.9 | -3.4 | -5.3 |
| GR | 6 | male | UI1 | -11.7 | -3.6 | -5.6 |
| GR | 8 | male | UI1 | -11.3 | -3.9 | -6.0 |
| GR | 9 | male | UI1 | -11.0 | -3.7 | -5.6 |
| GR | 10 | male | UI1 | -7.7 | -3.2 | -5.0 |
| GR | 11 | male | UI1 | -8.8 | -3.8 | -5.8 |
| GR | 12 | female | UI1 | -8.6 | -4.1 | -6.3 |
| GR | 13 | male | UI1 | -10.5 | -3.2 | -5.0 |
| GR | 15 | male | UI1 | -10.5 | -4.3 | -6.5 |
| GR | 18 | male | UI1 | -10.8 | -2.2 | -3.6 |
| GR | 19 | female | UI1 | -10.0 | -3.3 | -5.1 |
| GR | 21 | female | UI1 | -10.4 | -4.1 | -6.2 |
| GR | 22 | male | UI1 | -11.4 | -2.8 | -4.4 |
| GR | 24 | male | UI1 | -10.5 | -3.2 | -4.9 |
| GR | 26 | female | UI1 | -6.7 | -4.5 | -6.8 |
| GR | 32 | female | UI1 | -10.5 | -3.3 | -5.1 |
| GR | 33 | female | UI1 | -10.1 | -4.0 | -6.1 |
| GR | 35 | male | UI1 | -9.9 | -4.0 | -6.1 |
| GR | 42 | male | UI1 | -9.9 | -3.7 | -5.7 |
| GR | 43 | male | UI1 | -9.7 | -4.1 | -6.2 |
| GR | 46 | female | UI1 | -8.4 | -2.9 | -4.5 |
| GR | 47 | male | UI1 | -10.2 | -4.0 | -6.0 |
| GR | 48 | female | UI1 | -10.6 | -3.5 | -5.4 |
| GR | 50 | male | UI1 | -8.6 | -2.8 | -4.5 |
| GR | 51 | female | UI1 | -8.4 | -3.6 | -5.5 |
| GR | 53 | male | UI1 | -8.3 | -3.7 | -5.6 |
| GR | 55 | female | UI1 | -10.4 | -3.2 | -5.0 |
| TO | 1 | female | UI1 | -8.3 | -3.2 | -4.9 |
| TO | 2 | male | UI1 | -8.2 | -4.6 | -6.9 |
| TO | 3 | male | UI1 | -6.7 | -3.9 | -6.0 |
| TO | 4 | male | UI1 | -7.4 | -4.4 | -6.7 |
| TO | 6 | female | UI1 | -6.2 | -3.3 | -5.2 |
| TO | 8 | male | UI1 | -9.0 | -4.2 | -6.3 |
| TO | 9 | female | UI1 | -6.8 | -3.2 | -5.1 |
| TO | 12 | male | UI1 | -11.7 | -3.9 | -5.9 |
| TO | 14 | female | UI1 | -8.6 | -3.6 | -5.5 |
| TO | 16 | male | UI1 | -10.1 | -2.7 | -4.4 |
| TO | 18 | female | UI1 | -7.4 | -3.1 | -4.9 |
| TO | 19 | female | UI1 | -7.0 | -3.0 | -4.7 |
| TO | 20 | male | UI1 | -9.7 | -2.7 | -4.2 |
| TO | 21 | male | UI1 | -7.1 | -3.7 | -5.6 |
| TO | 22 | female | UI1 | -9.1 | -3.1 | -4.9 |
| TO | 24 | female | UI1 | -7.9 | -4.0 | -6.0 |
| TO | 25 | female | UI1 | -8.4 | -3.6 | -5.5 |
| TO | 26 | male | UI1 | -9.7 | -2.7 | -4.3 |
| TO | 27 | male | UI1 | -11.6 | -3.8 | -5.9 |
| TO | 28 | male | UI1 | -5.5 | -2.0 | -3.3 |
| TO | 29 | female | UI1 | -6.9 | -3.4 | -5.3 |
| TO | 30 | female | UI1 | -11.6 | -2.8 | -4.5 |
| TO | 31 | female | UI1 | -7.5 | -3.8 | -5.8 |
| TO | 32 | female | UI1 | -8.6 | -3.8 | -5.8 |
| TA | 1 | male | UI1 | -6.8 | -4.2 | -6.4 |
| TA | 2 | male | UI1 | -6.7 | -3.7 | -5.7 |
| TA | 3 | female | UI1 | -7.4 | -3.9 | -5.9 |
| TA | 4 | male | UI1 | -7.4 | -3.9 | -8.0 |
| TA | 5 | female | UI1 | -7.2 | -4.3 | -6.5 |
| TA | 6 | female | UI1 | -7.4 | -4.2 | -6.4 |
| TA | 7 | male | UI1 | -6.5 | -4.7 | -7.1 |
| TA | 8 | female | UI1 | -6.8 | -5.0 | -7.3 |
| TA | 9 | female | UI1 | -8.3 | -3.9 | -6.0 |
| TA | 10 | male | UI1 | -8.1 | -3.8 | -5.7 |
| TA | 11 | male | UI1 | -7.3 | -5.2 | -7.7 |
| TA | 12 | male | UI1 | -6.7 | -4.6 | -6.9 |
| TA | 13 | male | UI1 | -7.7 | -3.9 | -6.0 |
| TA | 14 | female | UI1 | -7.9 | -3.8 | -5.8 |
| TA | 15 | female | UI1 | -7.0 | -3.8 | -5.9 |
| TA | 16 | female | UI1 | -8.5 | -4.5 | -6.7 |
| TA | 17 | female | UI1 | -5.8 | -5.0 | -7.5 |
| TA | 18 | male | UI1 | -7.0 | -4.1 | -6.2 |
| TA | 19 | male | UI1 | -6.3 | -5.3 | -7.8 |
| TA | 20 | female | UI1 | -9.0 | -3.5 | -5.4 |
| BA | 22 | male | UI1 | -8.8 | -3.3 | -5.1 |
| BA | 66 | female | UI1 | -9.2 | -3.8 | -5.8 |
| BA | 118 | female | UI1 | -9.5 | -5.2 | -7.7 |
| BA | 180 | female | UI1 | -7.6 | -4,0 | -6.1 |
| BA | 187 | male | UI1 | -8.6 | -4.3 | -6.5 |
| BA | 247 | female | UI1 | -10.8 | -4.8 | -7.2 |
| BA | 258 | male | UI1 | -9.8 | -4.5 | -6.8 |
